# Structure and Physicochemical Properties of Malate Starches from Corn, Potato, and Wrinkled Pea Starches

**DOI:** 10.3390/polym11091523

**Published:** 2019-09-19

**Authors:** Miaomiao Shi, Yue Jing, Liuzhi Yang, Xianqing Huang, Hongwei Wang, Yizhe Yan, Yanqi Liu

**Affiliations:** 1 School of Food and Biological Engineering, Zhengzhou University of Light Industry, Zhengzhou 450002, China; chengzi3090@126.com (M.S.); jy133456jy@163.com (Y.J.); 2008040@zzuli.edu.cn (L.Y.); hwwang@zzuli.edu.cn (H.W.); 2 Collaborative Innovation Center of Food Production and Safety, Zhengzhou 450002, China; 3 Henan Key Laboratory of Cold Chain Food Quality and Safety Control, Zhengzhou 450002, China; 4 College of Food Science and Technology, Henan Agricultural University, Zhengzhou 450002, China; hxq8210@126.com

**Keywords:** malic acid, starch, structure, property

## Abstract

In this study, corn, potato, and wrinkled pea starches were esterified with malic acid under high temperatures for different lengths of time. The degree of substitution (DS), granule morphology, crystal structure, gelatinization properties, and the digestibility of the malate starch were investigated. Fourier transform infrared spectroscopy (FT–IR) suggested that the malate starch showed a new infrared absorption peak near 1747 cm^−1^, indicating the occurrence of an esterification reaction. With an increasing treatment time, the degree of substitution (DS) of the malate starch displayed an increasing trend. Scanning electron microscopy (SEM) demonstrated a significant change in the surface structure of the starch granules. X-ray diffractometry (XRD) reflected that the crystal structure of the malate starches was destroyed. The thermogravimetric (TG) curves showed that the maximum heat loss rate of the malate starch was ahead of that of native starch, which caused the decreased degree of crystallinity. These properties of malate starch could allow it to be used for the purpose of starch modification to produce resistant starch and to provide new applications for starch.

## 1. Introduction 

Starch is an important source of energy for the human diet and an important component of food [1]. It comes mainly from the roots, stems and seeds of cereals and potatoes [2]. Commercialized starches include native starches and modified starches. In recent years, the research focus on food grade organic acid in starch function improvement has gradually increased. Recently, research on the reaction of starch and organic acids to manufacture resistant starch in high yield has been increasingly favored by domestic and overseas scholars. Because resistant starch is low in calories and has high levels of dietary fiber, it has become a research hotspot in functional foods. Xie [3] used citric acid to react with native corn starch (140 °C, 7 h) to obtain 68.3% resistant starch. During this reaction, the hydroxyl group on the glucose ring in the starch chain reacted with citric acid to form a crosslinked structure derivative with a high percentage of resistant starch (RS). Hung [4] used citric acid, lactic acid, and acetic acid to react with waxy rice starch by a heat-moisture treatment (HMT) method, which increased the content of resistant starch from 30.1% to 39.0%. The content of RS was significantly higher than that of native starch. Luo studied the acetylation of three different amylose content corn starches in sodium hydroxide solution (20%, w/w) as a catalytic aqueous solution and investigated the structural differences in the resulting acetylated starch derivatives [5].

Esterified starches have various applications in the field of food processing, such as in thickeners, additives, stabilizers, emulsifiers, flavoring ingredients, and binders for drying foods. There are also nonfood uses, for instance, as fillers, super disintegrants, hot melt adhesives, coatings, etc. [6]. In the medical field, esterified starches are also very useful as controlled release agents. A product of starch and fatty acids with a certain chain length could help maintain human colon function and prevent colon disease [1]. Malic acid is a healthy and nonharmful food additive. It has a soft taste and is easily soluble in many organic solvents. It is normally stable and belongs to the polycarboxylic acids in chemical structure, as does citric acid. As a food additive, malic acid has been used in food preservation, deodorization, color retention, and salt reduction, but there have been few reports on it when used as a starch modifier.

Our previous research explored the reaction mechanisms of wrinkled pea starch and malic acid to produce resistant starch as measured through nuclear ^13^C CP/MAS NMR spectra, FTIR, etc., and simultaneously investigated the effect of HMT on the properties of esterified starch [7]. Corn, potato, and wrinkled pea starches belong to different A, B, and C crystal types, respectively. Starches of different crystal type exhibit different properties after esterification, which was meaningful for our research. It was the purpose of this paper to explore which starch was more sensitive to malic acid to give a high level of resistant starch.

In this experiment, corn, potato, and wrinkled pea starches were esterified with malic acid under high temperatures for different lengths of time. The structure, degree of substitution, physicochemical, and in vitro digestibility of malate starch were studied. The preparation of malate starch could achieve the purpose of starch modification to produce resistant starch and provide new applications for starch.

## 2. Materials and methods

### 2.1. Materials

Corn starch was purchased from Qinhuangdao Lihua Starch Co., Ltd. (Qinhuangdao, China) with 0.3% protein, 0.2% ash, 10.9% moisture, 23.2% amylose, and a little fat. Potato starch was produced by Weston Company (Qinghai, China), and it contained 0.1% protein, 0.3% ash, 15.8% moisture, 21.6% amylose, and a little fat. Wrinkled pea seeds (wild variety) were obtained from the farmers of Gansu Province in China. The wrinkled pea starch had 0.4% protein, 0.1% ash, 10.0% moisture, 30.8% amylose, and a little fat. Malic acid was acquired from Fuchen Chemical Reagent Factory (Tianjin, China). Pancreatin from porcine pancreas (P7545, 8×USP) and amyloglucosidase from *Aspergillus niger* (A7095, 300 U/mL) were purchased from Sigma-Aldrich Chemical Co. (St. Louis, MO, USA). Glucose oxidase–peroxidase (GOPOD) assay kits were obtained from Megazyme International Ireland Ltd. (Wicklow, Ireland). All other chemicals and reagents were of analytical grade.

### 2.2. Preparation of Malate Starch Samples 

The reaction of starch and malic acid was carried out following the method of Klaushofe [8] with appropriate modifications. Starch (25%, w/v) was dispersed into malic acid solution (20%, w/v), and the pH of the starch slurry was adjusted to 3 with sodium hydroxide solution (10 mol/L). The mixture was allowed to stand at room temperature for 12 h to allow full penetration of starch and malic acid. The starch slurry was kept in an air oven at 45 °C for a sufficient time to achieve a moisture content of less than 10%. The sample was ground, crushed, and put into a hydration reactor at 130 °C for 2 h, 3 h, 4 h, or 5 h. The unreacted malic acid was washed away with deionized water several times. The starch sample was dried at room temperature. Finally, the starch was crushed, and passed through a 100 mesh standard sieve to obtain the malate starch.

### 2.3. Determination of the Degree of Substitution (DS) 

The DS of the malate starch was determined by the method of Kweon [9]. The DS was calculated from the reactive group (2-hydroxysuccinyl) of malic acid. Malate starch (1 g, dry weight) was added into deionized water (10 mL), and then two drops of phenolphthalein indicator solution was added. The sample was titrated to the associated reddish color with NaOH (0.1 mol/L). NaOH (5 mL, 0.45 mol/L) was added into the mixture and stirred for 60 min. Excess sodium hydroxide was neutralized with a standard hydrochloric acid solution (0.2 M). Natural starch was used as a blank test. The results were calculated as follows [7]:

Mass fraction of malic acid substituents A:

(1) $$A\%=\frac{V_{0}{-V}_{1}\times C\times M\times 100}{W\times 1000}$$

DS of malate starch:

(2) $$\mathrm{DS}=\frac{162\times A}{100M-\left(M-1\right)A}=\frac{162\times A}{11700-116A}$$

V_0_—the volume of HCl solution consumed by the blank, mL;
V_1_—the volume of HCl solution consumed by the sample, mL;
C—the exact concentration of the standard titration solution of hydrochloric acid, mol/L;
M—the molar content of the 2-hydroxysuccinyl group, g/mol (M(C_4_H_5_O_4_) = 117);
W—the mass of the sample, g;
162—the relative molecular mass of glucosyl;
1000—conversion factor.

### 2.4. Scanning Electron Microscopy (SEM) 

After the starch was sprayed with gold, the SEM image was taken using a scanning electron microscope (PhilipsXL-3, HITACHI, Tokyo, Japan) at 20 KV [10]. 

### 2.5. X-Ray Diffraction (XRD) 

The XRD patterns of starch samples were obtained by an X-ray diffractometer (Burker D8, Karlsruhe, German) at 3 kV and 20 mA with 0.1542 nm Cu Kα radiation (Ni filter). The scanning range was 2θ values of 5–35° at a 0.04° step size and with a scanning rate of 4°/min [11]. The relative crystallinity was obtained by using the following formula:

(3) $$\mathrm{Crystallinity}\;(\%)=\frac{I_{c}}{{(I}_{a}{+I}_{c})}\times 100$$

where I_a_ and I_c_ are the amorphous and crystalline area, respectively, on the X-ray diffractogram.

### 2.6. Fourier Transform Infrared Spectroscopy (FT–IR) 

The sample and the required KBr were dried in an air oven at 40–50 °C for 4–5 h to avoid the influence of moisture before testing. FT–IR analysis of the malate starches were executed over a wave number range of 4000–400 cm^−1^ with a resolution of 4 cm^−1^ on an FT–IR instrument (Vertex70, Karlsruhe, German) The samples were scanned 64 times using the transmission method. Finally, the average value was obtained [12].

### 2.7. Thermogravimetric (TG) Analysis 

The thermal stability of the malate starch sample was performed using a differential thermal analyzer (Diamond TG/DTA-A, Norwalk, America). Starch (3 mg, dry weight) was heated in an aluminum oxide pan from 30 to 600 °C with a 10 °C /min heating rate and 40 mL/min N_2_ gas flow [13]. The temperatures at which the malate starch began to lose and completely lost water can be seen from the TG curve.

### 2.8. In Vitro Starch Digestion

In vitro digestibility of starch was evaluated using the method of Englyst [14], as modified by Du [15]. Starch (1 g, dry weight) with 20 mL of sodium acetate buffer (0.1 mol/L, pH 5.2) was gelatinized (100 °C) for 30 min, and then cooled down to room temperature. An enzyme suspension (5 mL, pancreatin and amyloglucosidase) was added to the starch solution. The mixture was shaken in a constant temperature (37 °C) water bath oscillator (SHZ-82, Jinyi Instrument Technology Co., Ltd, Changzhou, China) at 150 r/min for 2 h. Aliquots (0.5 mL) were taken at different times (20 and 120 min) and mixed with 70% ethanol (20 mL) to stop enzymatic digestion. These solutions were centrifuged (3000 r/min, 10 min), and the hydrolyzed glucose content of the supernatants were measured using the GOPOD kit at 510 nm. The rapidly digested starch (RDS), slowly digested starch (SDS), and resistant starch (RS) contents were calculated using the following equations [16]:

(4) $$\mathrm{RDS}\;\left(\%\right)=\frac{{(\mathrm{Glucose}}_{20\min}-\mathrm{FG})\times 0.9}{\mathrm{TS}}\times 100\%$$

(5) $$\mathrm{SDS}\;\left(\%\right)=\frac{{(\mathrm{Glucose}}_{120\min}{-\mathrm{Glucose}}_{20\min})\times 0.9}{\mathrm{TS}}\times 100\%$$

(6) RS (%)=1−RDS%−SDS%

where TS represents the weight of the starch sample; Glucose_20min_ and Glucose_120min_ represent the amount of glucose released within 20 and 120 min, respectively and FG represents the glucose content (free glucose) in the sample before hydrolysis.

### 2.9. Statistical Analysis

The data reported were the averages of triplicate determinations and the results were represented as the mean values ± SD (standard deviation). Analysis of the data was performed through variance analysis (ANOVA) and significance analysis was done by Duncan’s multiple range tests. Significance analysis was performed using SPSS 21.0. The level of significance was set at *p* < 0.05. All the graphs were obtained using Origin 6.1 software.

## 3. Results and Analysis

### 3.1. Degree of Substitution (DS) Analysis of the Malate Starch Samples

**Figure 1:**
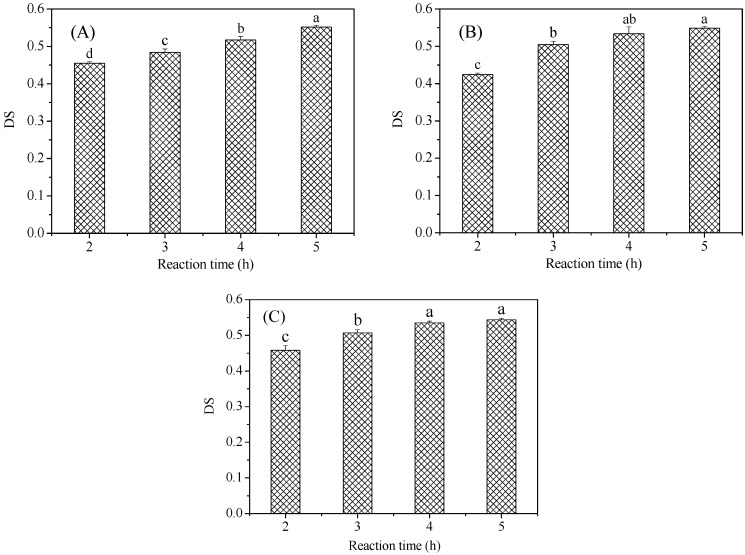
DS (degree of substitution) of malate starch samples: (**A**) Corn starch, (**B**) Potato starch, (**C**) Pea starch. Different letters in the same chart represent significant differences between different samples (*p* < 0.05).

Starches with different crystal types were reacted with malic acid for different dry heat times. Reaction time had a great impact on the DS of the malate starch. The DS values of malate starches prepared over different reaction times are shown in Figure 1. As the reaction time increased, the DS of the malate starch increased significantly. When the dry heat treatment time increased from 2 h to 5 h, the DS of corn, potato, and wrinkled pea malate starch samples gradually increased from 0.455, 0.424, and 0.458 to 0.552, 0.549, and 0.544, respectively. This was because the increased dry heat treatment time could promote malic acid more easily when combined with starch, which could increase the DS and accelerate the reaction rate. In addition, researchers indicated that the DS value of crosslinked rice starch with citric acid was shown to increase along with the concentration [17]. Within 3 h, the esterification reactions of potato and wrinkled pea starch were basically completed, and a prolonged reaction time after this point had little effect on the esterification reaction. This was consistent with the results of Zuo [18]. After 3 h, the reaction efficiencies of potato and pea starch tended to balance. Thereafter, the effect of reaction time on the DS was not obvious. For corn starch, the DS was increased after 3 h. This may be due to differences in the structure and properties in different crystalline starches.

### 3.2. Scanning Electron Microscopy (SEM) of the Malate Starch Samples

**Figure 2:**
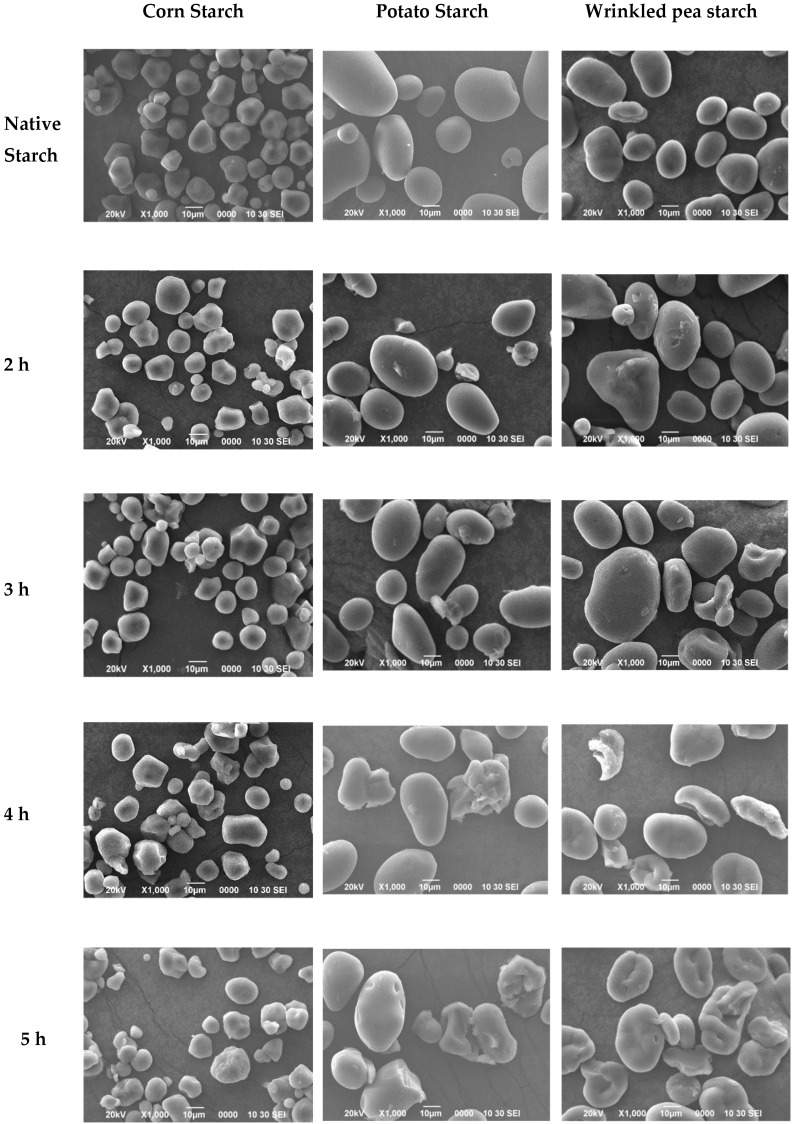
Scanning electron microscopy of native starch and malate starch heat treated for different lengths of time (1000×).

The granular morphologies of native starches and malate starches treated for different reaction times are shown in Figure 2. It can be seen that the native corn starches were a polygonal shape with a plurality of planes and edges on the surface. However, after malic acid treatment, the center of the corn starch exhibited depressions and deformations. The formation of some spots and cracks on the surfaces of wheat starch granules under citric acid treatment was also observed by Majzoobi [19]. Some starch granules adhered to each other. Deformation of esterified corn starch granules with a lower DS was reported by López-Rubio [1]. It can be seen from Figure 2 that the native potato starch granules had a smooth surface with an oval shape and a complete granule. Most of the potato starch granules were intact as the esterification reaction progressed, however, the partial granules exhibited depressions and ruptures on the surface after 4 h and 5 h of dry heat treatment time. This was consistent with previous studies on heat–moisture-treated tapioca starch by Andrade [20], who also found depressions and damage on the surfaces of starch granules. Fornal [21] observed jagged areas and ring shapes in the surface of commercial food grade esterified potato starch granules. Similar shapes were found on potato starch granules after a hydroxypropylation reaction [22]. Wrinkled pea starches were mostly flat and ellipsoidal in shape with slight wrinkles on the surface. The average granule sizes of native wrinkled pea starches were 10–40 μm. After the esterification reaction, more pits and wrinkles appeared on the surfaces of wrinkled pea starch granules. The granules retained their size and shape, indicating that the esterification reaction mainly happened on the surface of the starch particle. Jing found that starch granules were more sensitive to esterification if they had high amylose content (≥50%) [23]. Observation of rice starch modified with citric acid showed that the esterification reaction did not destroy the integrated structure of the starch granule [24]. After 130 °C dry heat treatment, some starch granules showed obvious changes. In the early stage of the dry heat treatment, the water inside the starch granules rapidly vaporized and volatilized. At the end of the treatment, the starch granules were rapidly cooled, which caused the surfaces of starch granules to collapse inward. Kawabata [25] also found that heat–moisture treatment caused depressions, cracks, and crosslinks on the surfaces of starch granules.

The surface of esterified starch was rougher than that of native starch, which was easier to gather together. The surface of starch granules becoming rough is a common feature of esterified starch. The aggregation of starch granules could be caused by the surface destruction of some starch granules during chemical modification [21]. It has been observed that the rise in starch granule roughness after the esterification reaction could increase the cross-linking surface area of starch granules, which could improve the adhesion of starch to starch through synthetic polymers [26]. These significant changes in starch granule morphology could have an influence on the properties of starch, which is significant for resistant starch production and application.

### 3.3. X-Ray Diffraction (XRD) of the Malate Starch Samples

**Figure 3:**
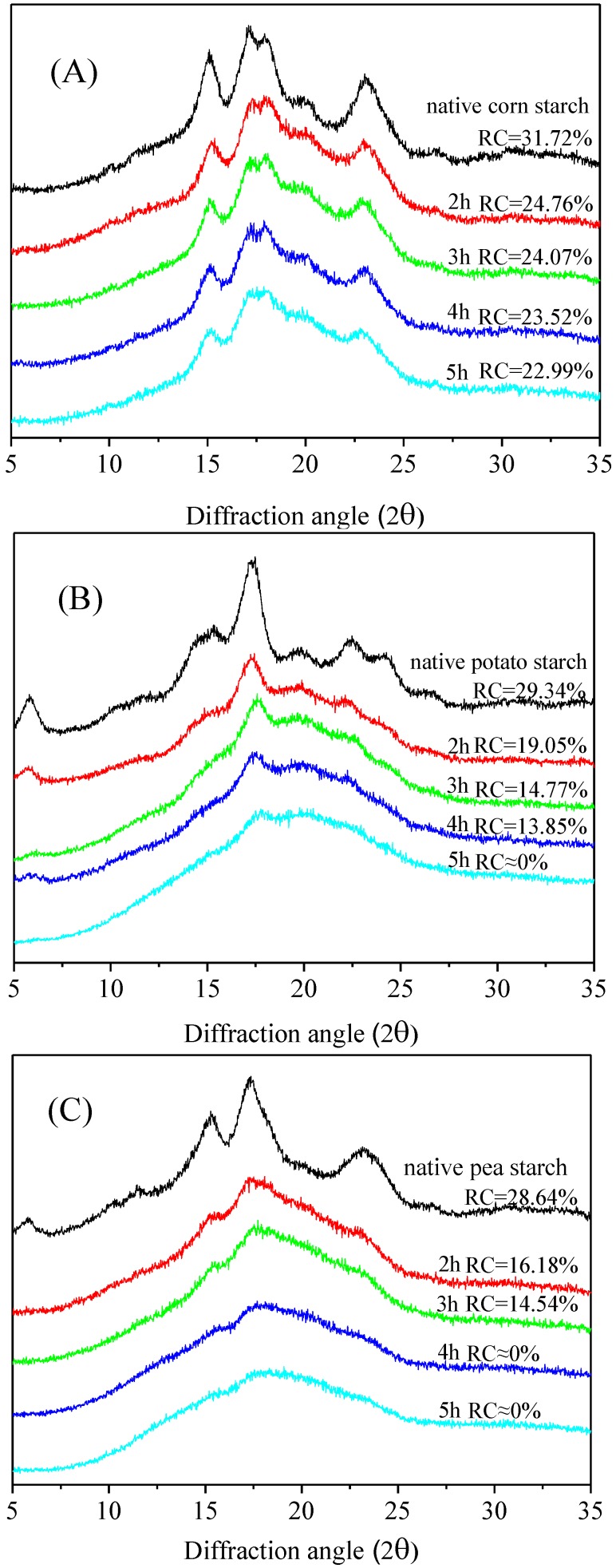
X-ray diffraction patterns of malate starch samples: (**A**) Corn starch, (**B**) Potato starch, (**C**) Pea starch.

The X-ray diffraction patterns of different crystalline native starches and malate starches treated for different times are shown in Figure 3. Native corn starch exhibited strong deflections at 2θ = 15.3°, 17.1°, 18.2°, and 23.1°, with a typical A-type crystalline pattern (Figure 3A). Native potato starch had major peaks at 2θ = 5.62°, 17.1°, 22.3°, and 24.0°, which are typical of the B-type crystalline pattern (Figure 3B). As shown in Figure 3C, native pea starch revealed a C-type crystalline pattern. Its peaks were present at 2θ = 5.75°, 15.3°, 17.3°, 18.2°, and 23.3°. The intensity of the characteristic peaks gradually decreased as the DS increased, indicating that the crystal structure of the native starch was destroyed [27]. This is because the esterification reaction led to damage of the double helix structure, and the crystalline region of the starch was destroyed [28]. The crystalline region of starch is mainly composed of amylopectin, which is a double helix formed by intermolecular hydrogen bonds. [29]. During an esterification reaction, when the hydrogen bonds between molecules are weakened, the starch crystallinity is reduced, which causes the destruction of the original ordered crystal structure [30]. With increasing dry heat treatment time, the crystal structures of the three types of starch were subjected to different degrees of damage. Extension of the dry heat treatment time caused most of the starch hydroxyl groups to be substituted. The order of destruction was pea starch > potato starch > corn starch. The crystalline types of the three starches were type A, type B, and type C, respectively. The structure and properties were different in the three starches. The degree of crystallinity (RC) of native wrinkled pea starch was 28.64%, whereas the RC of wrinkled pea starch treated for 5 h was approximately equal to zero. The same phenomenon was also found in potato starch treated for 5 h. Therefore, this indicated that the malate starch showed amorphous properties [31]. This was consistent with the structures of the starch granules observed by previous scanning electron microscopy.

### 3.4. Fourier Transform Infrared Spectroscopy (FT–IR) of the Malate Starch Samples

**Figure 4:**
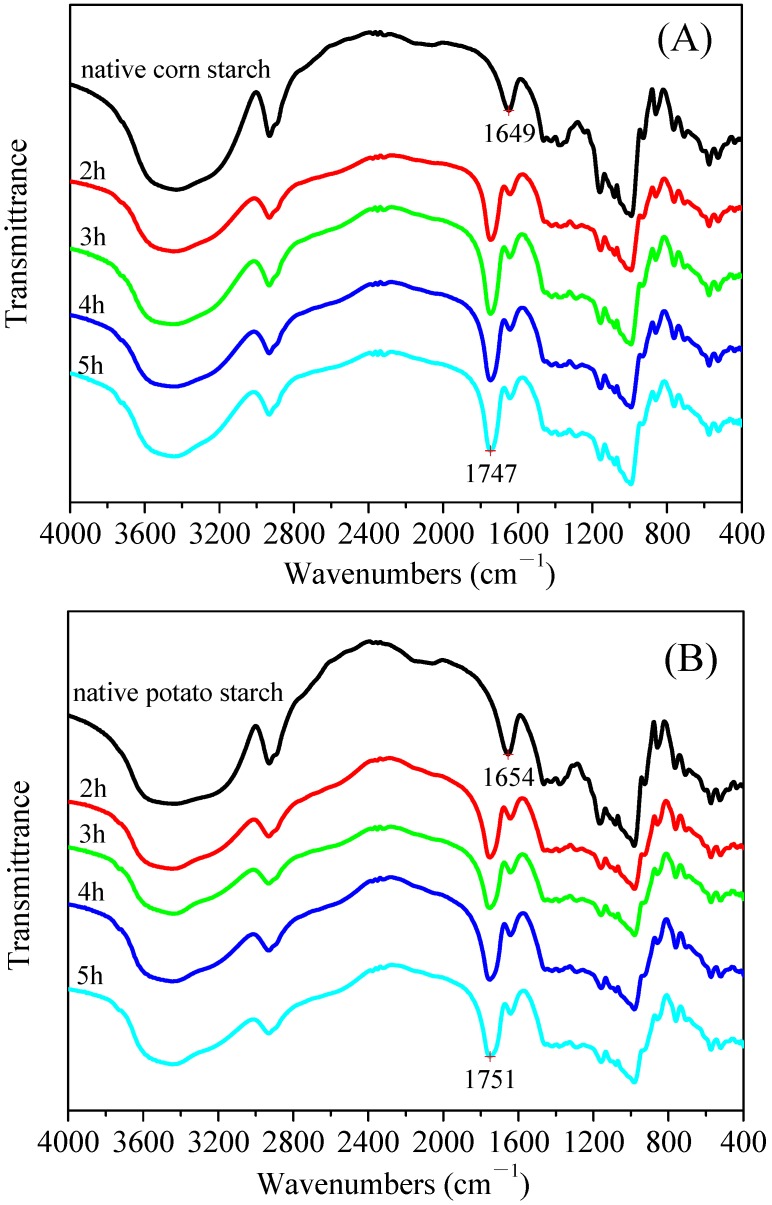

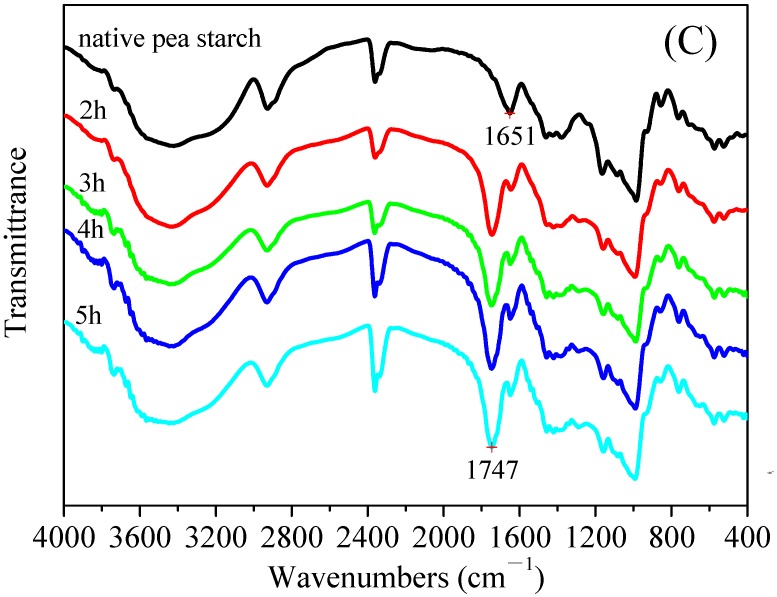
Fourier Transform Infrared Spectroscopy spectra of malate starch samples: (**A**) Corn starch, (**B**) Potato starch, (**C**) Pea starch.

Infrared spectroscopy is an important method used to observe any changes in the functional groups on the starch molecules. The resulting infrared spectra of the native starches and malate starches are shown in Figure 4. Traditionally, FT–IR spectroscopy of esterified starch has been used to provide qualitative evidence of the esterification reaction [32]. FT–IR showed that malate starches from the different crystalline starches presented obvious new characteristic peaks neighboring 1747 cm^−1^, which was related to the new ester linkages between carboxyl groups of malic acid and hydroxyl groups of starch [33]. The broadband peak centered around 3400 cm^−1^ was formed by the stretching vibration of the starch hydroxyl group. The decrease in the broadband peak indicated that the hydroxyl group was gradually replaced by the ester group. The data further confirmed that there was an esterification reaction between starch and malic acid. The new characteristic peaks neighboring 1747 cm^−1^ signal were weak in the malate starch samples with the 2 h dry heat reaction time level, however, with the extension of the dry heat reaction time, the intensity became stronger. The effect was most significant for malate starch treated for 5 h, which indicated that an appropriate dry heat treatment time could promote the esterification reaction and increase the DS.

### 3.5. Thermogravimetric (TG) Analysis of the Malate Starch Samples

**Figure 5:**
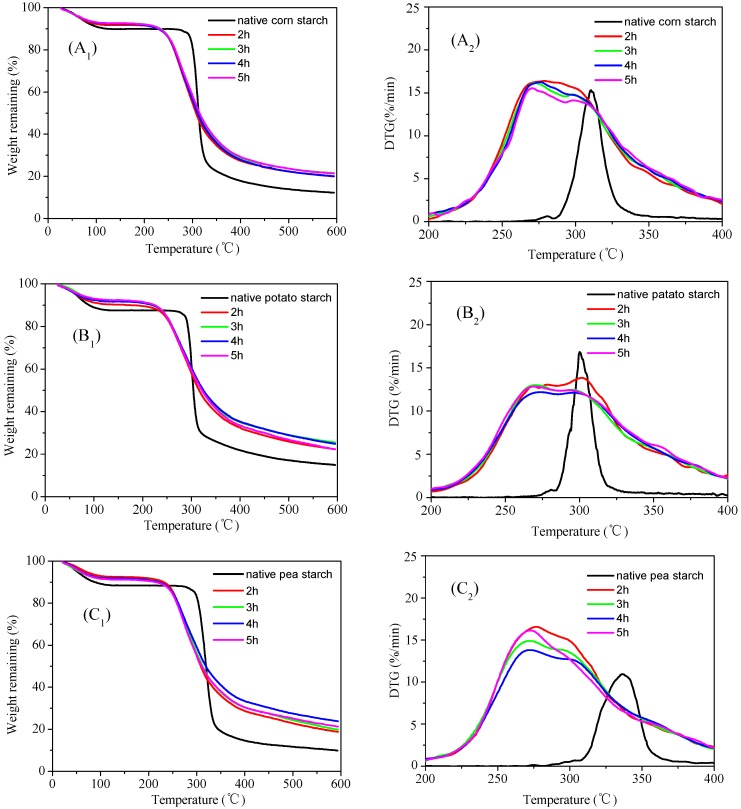
Thermal gravimetric analysis and derivative thermogravimetric analysis (TG/DTG) image of malate starch samples: (**A_1_**) Corn starch TG, (**A_2_**) Corn starch DTG, (**B_1_**) Potato starch TG, (**B_2_**) Potato starch DTG, (**C_1_**) Pea starch TG, (**C_2_**) Pea starch DTG.

Thermogravimetric analysis was used to study the thermal stability and composition of materials [34]. When the samples underwent heating, cooling, and isothermal hold, TG spectra were used to detect mass loss. During heating, it can accurately measure the sample mass if the sample releases gas or if physical changes occur [35]. To investigate the thermal stability of malate starch under high temperatures for different lengths of time, thermogravimetry was used to analyze malate starch samples. Figure 5 shows the results of thermal gravimetric analysis and derivative thermogravimetric analysis (TG/DTG) for native and malate starches. As in the case of biomass pyrolysis, for each heating rate, the initial decrease in weight was due to water release. Due to the evaporation of water and the low molecular-weight substances of the malate starch, the TG curves (A_1_/B_1_/C_1_) showed an initial peak (first mass loss) at 70–130 °C. The mass was stable until 200 °C and the DTG curves (A_2_/C_2_) showed a second peak at 250–300 °C, which indicated prominent mass loss for the corn and wrinkled pea malate starches. The DTG curves (B_2_) showed a second peak at 300–325 °C for potato malate starch. The main mass loss was caused by the elimination of polyhydroxyl groups, accompanied by depolymerization and decomposition [36]. This also suggested that malic acid glycoside had been successfully esterified to starch molecular chains. The derivative thermogravimetry (DTG) curves revealed that the maximum heat loss rate of malate starch was ahead of that of native starch, which caused the decreased degree of crystallinity. It was easier to destroy the malate starch chains during heating with the lower crystallinity. Cyras [36] found that the esterified starch had lower thermal stability than the native starch owing to the low crystallinity. The x-ray diffraction data showed that the esterification reaction destroyed the starch crystallization region, and the starch molecule density was decreased, so the malate starch was easily decomposed during heating. At the same time, it was also found that the starch dissolution process increased after esterification. This was because the esterification reaction of malic acid and starch caused degradation of acidified starch. In addition, the esterification substitution was nonuniform, which led to a prolonged dissolution process.

### 3.6. Digestibility Properties of the Malate Starch Samples

The digestibility properties of the malate starch are presented in Table 1. For all the samples, as the dry heat treatment time increased, the RDS and SDS contents of the malate starches decreased significantly. The RS contents of the malate starches were higher than those of the native counterparts, and they increased with increasing dry heat treatment time. As the dry heat treatment time increased from 2 h to 5 h, the RS contents of corn, potato, and wrinkled pea malate starch samples gradually increased from 15.22, 21.18, and 19.53 to 91.97, 90.49, and 95.23%, respectively. Similar findings have been reported in cassava and potato esterified starches by Hung [4]. With the extension of the dry heat treatment time, the hydroxyl groups in starch molecules were replaced by malate groups. This led to the formation of the malate starch with a crosslinked structure, which prevented the hydrolysis of the amylase. During the 2 h dry heat treatment, the RS content of wrinkled pea starch (71.56%) was higher than that of corn (63.61%) and potato (62.96%) starches. Similar results were found in the 3 h and 4 h dry heat treatment. Xie [3] believed that the difference in chemical reactions was mainly due to the differences in starch crystal type and molecular structure. Thus, the different RS content in wrinkled pea starch from those in corn and potato starches might have been due to the changes in the crystalline structure indicated in the XRD and FT–IR results analysis. These results showed that the longer the heat treatment time, the greater the amount of RS. Li [9] found that the increase in resistant starch content was due to the formation of starch citrate diester during the reaction of starch and citric acid under high temperature. The esterification reaction of starch and malic acid was similar to that of starch and citric acid. Therefore, the increased RS contents may have been due to the interaction of malic anhydrides with starch during dry heat treatment. After 3 h, the RS contents of malate starches tended to be stable, which was consistent with the DS results analysis.

## 4. Conclusions

The experiment above demonstrated successful esterification reactions of starch and malic acid by the dry method. The esterification reactions of malic acid with different crystalline starches caused significant changes in the structure, morphology, and gelatinization properties of malate starch. The different crystalline malate starches presented obvious characteristic peaks around 1747 cm^−1^—the C=O absorption shock peak—reflecting that the starch was esterified with malic acid. With increased dry heat treatment times, the DS of malate starch showed an overall upward trend, and the starch surface structure changed significantly. Depressions, cracks, and crosslinks appeared on the surfaces of starch granules. The esterification of starch destroyed the crystal structure, especially in pea starch. The amorphous properties of malate starch were revealed. The heat loss rates and thermal stability of the malate starches were lower than those of native starch, and its dissolution process became longer. The RS contents in the malate starch samples increased significantly. Therefore, the esterification reaction of starch from different sources and malic acid by the dry heat method can effectively increase the content of resistant starch.

## Figures and Tables

**Table 1:** Nutritional fractions of native and malate starch.

Samples	RDS (%)	SDS (%)	RS (%)
Native corn Starch	79.27 ± 3.83 ^a†^	5.50 ± 4.18 ^c^	15.22 ± 3.53 ^h^
Malate corn starch-2h	33.63 ± 1.10 ^d^	2.75 ± 1.34 ^d^	63.61 ± 2.18 ^f^
Malate corn starch-3h	19.52 ± 0.66 ^f^	2.22 ± 0.28 ^d^	78.26 ± 0.93 ^d^
Malate corn starch-4h	9.61 ± 0.77 ^h^	0.71 ± 0.43 ^d^	89.68 ± 0.43 ^b^
Malate corn starch-5h	7.11 ± 0.61 ^ij^	0.92 ± 0.67 ^d^	91.97 ± 0.20 ^ab^
Native potato Starch	42.73 ± 1.36 ^c^	36.10 ± 3.32 ^a^	21.18 ± 4.32 ^g^
Malate potato starch-2h	35.74 ± 1.09 ^d^	1.30 ± 0.19 ^d^	62.96 ± 0.91 ^f^
Malate potato starch-3h	16.37 ± 0.38 ^g^	1.27 ± 0.47 ^d^	82.35 ± 0.73 ^c^
Malate potato starch-4h	14.68 ± 0.20 ^g^	1.57 ± 0.52 ^d^	83.75 ± 0.71 ^c^
Malate potato starch-5h	8.71 ± 0.68 ^hi^	0.80 ± 0.50 ^d^	90.49 ± 0.33 ^b^
Native pea Starch	66.36 ± 2.49 ^b^	14.12 ± 2.25 ^b^	19.53 ± 4.43 ^g^
Malate pea starch-2h	26.88 ± 0.51 ^e^	1.56 ± 0.29 ^d^	71.56 ± 0.80 ^e^
Malate pea starch-3h	6.20 ± 0.32 ^jk^	0.87 ± 0.09 ^d^	92.93 ± 0.31 ^ab^
Malate pea starch-4h	5.05 ± 0.27 ^jk^	0.79 ± 0.47 ^d^	94.16 ± 0.48 ^a^
Malate pea starch-5h	4.02 ± 0.25 ^l^	0.74 ± 0.51 ^d^	95.23 ± 0.27 ^a^

Values are expressed as means ± SD (n = 3). ^†^ Different letters in the same column indicate a significant difference at P < 0.05 within the same starch. The data are presented as the mean ± standard deviation. RDS: rapidly digestible starch; SDS: slowly digestible starch; RS: resistant starch.
